# A comparative analysis exposes an amplification delay distinctive to SARS-CoV-2 Omicron variants of clinical and public health relevance

**DOI:** 10.1080/22221751.2022.2154617

**Published:** 2022-12-24

**Authors:** K.L. Brown, A. Ceci, C. Roby, R. Briggs, D. Ziolo, R. Korba, R. Mejia, S.T. Kelly, D. Toney, M.J. Friedlander, C.V. Finkielstein

**Affiliations:** a Virginia Tech Carilion School of Medicine, Virginia Tech, Roanoke, VA, USA; b Molecular Diagnostics Laboratory, Fralin Biomedical Research Institute at VTC, Virginia Tech, Roanoke, VA, USA; c Integrated Cellular Responses Laboratory, Fralin Biomedical Research Institute at VTC, Virginia Tech, Roanoke, VA, USA; d Fralin Biomedical Research Institute at VTC, Virginia Tech, Roanoke, VA, USA; e Department of Biological Sciences, Virginia Tech, Blacksburg, VA, USA; f ZC Lab Services, Greenacreas, FL, USA; g Molecular Detection and Characterization, Department of General Services, Division of Consolidated Laboratory Services, Richmond, VA, USA; h Center for Zoonotic and Arthropod-borne Pathogens, Virginia Tech, Blacksburg, VA, USA

**Keywords:** Omicron variant, SARS-CoV-2, Delta variant, RT-qPCR, diagnosis, mismatch, amplification efficiency, COVID-19

## Abstract

Mutations in the SARS-CoV-2 genome may negatively impact a diagnostic test, have no effect, or turn into an opportunity for rapid molecular screening of variants. Using an in-house Emergency Use Authorized RT-qPCR-based COVID-19 diagnostic assay, we combined sequence surveillance of viral variants and computed PCR efficiencies for mismatched templates. We found no significant mismatches for the *N*, *E*, and *S* set of assay primers until the Omicron variant emerged in late November 2021. We found a single mismatch between the Omicron sequence and one of our assay’s primers caused a > 4 cycle delay during amplification without impacting overall assay performance.

Starting in December 2021, clinical specimens received for COVID-19 diagnostic testing that generated a Cq delay greater than 4 cycles were sequenced and confirmed as Omicron. Clinical samples without a Cq delay were largely confirmed as the Delta variant. The primer-template mismatch was then used as a rapid surrogate marker for Omicron. Primers that correctly identified Omicron were designed and tested, which prepared us for the emergence of future variants with novel mismatches to our diagnostic assay's primers. Our experience demonstrates the importance of monitoring sequences, the need for predicting the impact of mismatches, their value as a surrogate marker, and the relevance of adapting one's molecular diagnostic test for evolving pathogens.

## Introduction

As the coronavirus disease-2019 (COVID-19) pandemic progressed over the last two years, the genome of severe acute respiratory syndrome coronavirus 2 (SARS-CoV-2) evolved into sequence variants that conferred to the virus fitness advantages impacting global health. As time passed, the accumulation of mutations in SARS-CoV-2 led to increasing transmission efficiency and virulence and the ability to partially evade the host immune response [1-3]. As a result, vaccines and antiviral drugs have become less effective and diagnostic tests less accurate for a specific variant’s detection; thus, it is imperative to continuously monitor the evolution of SARS-CoV-2 [4].

From a public health perspective, the continuous accumulation of polymorphisms in the SARS-CoV-2 genome needs to be monitored for surveillance but also to rapidly mitigate and contain the spread of the virus. Accordingly, actionable information arising from genomic surveillance can be channeled into public health strategies that impact morbidity and mortality. In today’s post-genomic world, genomic surveillance exploits the power of next-generation sequencing and relies on publicly available global sequence repositories that emerged during the COVID-19 pandemic as well as existing genomic networks [5,6]. As a result, sequence data not only provides a temporal and geographical distribution of variants but, importantly, helps to predict antigenicity and phenotypic changes that could impact a variant’s detection and treatment.

To support public health efforts in mitigating the spread of SARS-CoV-2 in rural Southwest Virginia, our laboratory developed a molecular diagnostic test that received Emergency Use Authorization from the U.S. Food and Drug Administration early in the pandemic [7]. That assay, which is still being used, has been implemented to screen almost a quarter of a million individuals in the region, and uses real-time reverse-transcription polymerase chain reaction (RT-qPCR) to amplify three distinct regions: the Spike (*S*), Envelope (*E*), and Nucleocapsid (*N*) of the SARS-CoV-2 RNA genome. Our diagnostic test was designed to detect the original strain of SARS-CoV-2 from Wuhan, China and its immediate variants, as the regions selected for its RT-qPCR primers lie in relatively stable regions of the viral genome [7]. A clinical specimen is reported positive for SARS-CoV-2 if at least two of the three target regions are amplified by the assay. Therefore, for a potential false-negative result to occur due to mutations, there would need to be template mutations that negatively impact PCR amplification for at least two of its three primer sets.

Since the implementation of our SARS-CoV-2 assay, regular sequence surveillance was set-up to analyze viral genomic sequences as they became available to monitor for mismatches under the assay primers that could impact the assay’s performance. Accordingly, assay primers could then be adjusted to ensure coverage of circulating sequence variants of SARS-CoV-2. Alternatively, the existence of changes in primer-probe efficiencies can be exploited, when possible, for the purpose of predicting circulating variants [8-10].

In this article, we report the identification of a single non-synonymous mutation 26270C > T (codon T9I) within the *E* gene of Omicron variants that impacts primer-template annealing, but not PCR amplification efficiency, which results in a Cq delay. Retrospective data analyses of samples collected since January 2021, show that this primer mismatch provides an amplification readout with Cq values greater than 4 cycle later compared to the Cq values of the *N* and *S* genes only in samples collected starting in mid-December 2021 at a time when Omicron was detected in our region. Between December 13, 2021 and January 31, 2022, we analyzed 4,619 positive clinical samples out of 17,744 total samples and performed amplicon Sanger sequencing or whole genome sequencing to identify the SARS-CoV-2 variant. Our results show a near perfect correlation between a Cq delay and the identification of the Omicron sequence among positives. Consequently, we propose that, as long as T9I remains a signature mutation among emerging variants and is the only mismatch in the *Ef* primer, the Cq delay can be used as a surrogate for rapid and cost-effective tracking of the prevalence of Omicron variants. Furthermore, this molecular tool provides a unique approach to rapidly diagnose Omicron in clinical samples in situations where resources for treatment are scarce and whole genome sequencing cannot be broadly implemented.

## Materials and methods

***Analysis of published SARS-CoV-2 sequences.*** Up to 10,000 SARS-CoV-2 sequences for each VOI and VOC were downloaded from GISAID (https://www.gisaid.org/). Preference was given to complete sequences excluding low coverage sequences. If less than 10,000 sequences were available, all sequences were included in the analysis. Sequences were aligned using the DECIPHER package in R studio as previously described [7]. Sequences with mixed bases under the primer binding regions or missing sequence information were excluded from the analysis. For each primer mismatch pattern found, PCR efficiency was predicted using the DECIPHER package [11]. Computer code is provided as Supplementary Data.

***Testing of synthetic Delta and Omicron RNA templates.*** Synthetic single-stranded RNA representing SARS-CoV-2 Delta (B.1.617.2, EPI_ISL_6841980, control 23, cat# 104533) and Omicron (B.1.1.529/BA.1, EPI_ISL_1544014, control 48, cat# 105204) were obtained from Twist Biosciences (South San Francisco, CA). A 1:5 serial dilution series in nuclease-free water was prepared and used as a template for amplification and detection of the Nucleocapsid (*N*), Envelope (*E*) and Spike (*S*) genes using our standard SARS-CoV-2 assay as described [7]. Supplementary Table 1 lists the primers used for amplification. Each dilution was tested by RT-qPCR in triplicate.

***Screening of clinical samples.*** Testing and implementation protocols were under Emergency Use Authorization by the Federal Drug Administration (EUA# 200383) and approved by the Institutional Review Boards of Virginia Tech (IRB# 20-852) and the Virginia Department of Health (IRB# 70046). Informed written consent was obtained from the participants for both sample collection and its use in research. Clinical specimens were collected from individuals suspected of having COVID-19 using nasopharyngeal swabbing (NP) by trained healthcare professionals.

From December 13, 2021 through January 31, 2022, 17,744 clinical specimens were submitted to Virginia Tech Schiffert Health Center’s Molecular Diagnostics Laboratory (Roanoke, VA) for SARS-CoV-2 testing. Roughly ninety-three percent of positive samples (4,289 out of 4,619) were subjected to whole genome sequencing or screened by rapid mutational analysis to determine their variant type.

***Rapid mutational analysis (RMA).*** Four-to-six target regions of the SARS-CoV-2 genome that span known mutations of Delta (6 mutations common to all subvariants) and Omicron (15, 17, 20, and 18 mutations for BA.1, BA.2, BA.4, BA.5, respectively) were amplified and sequenced using the standard Sanger method. Briefly, clinical samples were diluted (1:20) and then amplified using the Power SYBR™ Green RNA-to-CT™ 1-Step Kit (Applied Biosystems) with a modified amplification program (45 cycles of 95°C 15 sec, 61°C 30 sec, and 72°C 45 sec). Primers were added at a final concentration of 300 nM. Amplification reactions were treated with ExoSAP-IT™ Express (Applied Biosystems) and submitted to Eurofins Genomics (Louisville, KY) for Sanger sequencing. Primers used for RMA amplification and sequencing were a combination of ARTIC V3/V4.1 primers [12] or custom primers designed using Primer Quest software (Integrated DNA Technologies) (Supplementary Table 2). A “suspected variant” call was made for each sample based on variant-defining mutations (https://outbreak.info/, covariants.org) identified within the sequenced regions of the SARS-CoV-2 genome.

***Whole genome sequencing (WGS) procedure and sequence analysis.*** cDNA was synthesized from selected positive RNA samples (Ct ≤ 34) using SuperScript IV Reverse Transcriptase (Thermofisher). PCR amplification was carried out using primers from the ARTIC nCoV-2019 Amplicon Panel, V4.1 (Integrated DNA technologies). Amplicons were barcoded using the plexWell™ 384 Library Preparation Kit (seqWell, MA) and sequenced using a MiSeq System (Illumina, CA).

SARS-CoV-2 sequencing analysis was accomplished using a combination of tools to determine quality assessment, alignment, variation calling, and variant assignment. The analysis tools were packaged into a Docker (https://www.linuxjournal.com/content/docker-lightweight-linux-containers-consistent-development-and-deployment) container built on DockerHub (https://hub.docker.com/). The image was based on the State Public Health Bioinformatics Workgroup Docker images and workflows (http://www.staphb.org/). The Docker container was pulled and converted into a Singularity (https://doi.org/10.5281/zenodo.5564905) image on the Rescale cloud platform (https://rescale.com/). Analysis via the Rescale cloud platform started by pulling data from the sequencing source using the Rescale cloud in the Design of Experiments (DOE) mode where every sample was treated as a parameter. This allowed for horizontal scaling to effectively give unlimited capacity through cloud scaling in a fixed amount of time.

Briefly, the analysis pipeline started by trimming the data using Trimmomatic (http://www.usadellab.org/cms/?page = trimmomatic) and then mapping against the SARS-CoV2-2 genome (MN908947.3 – Wuhan variant) using Minimap2 (https://academic.oup.com/bioinformatics/article/34/18/3094/4994778). The mapping files were converted and sorted using Samtools (http://www.htslib.org/) to produce a final sorted bam file. Summary statistics were also extracted from the bam file using Samtools. Following mapping, a consensus sequence was created using iVAR (https://hpc.nih.gov/apps/iVar.html)*.* iVAR takes as input the alignment file and a primer bed file containing the primer pairs used to create the sequencing library (Illumina Arctic V3/V4.1). Samtools was used once more to sort, index the masked consensus, and create a mpileup file with mapping statistics calculated and collected. Variations were called using the mpileup file and iVAR. Lastly, Pangolin (https://cov-lineages.org/) was used to type the SARS-CoV-2 strain. All WGS sequences were deposited in the GISAID (https://www.gisaid.org/) and NCBI (https://ncbi.nlm.nih.gov) SARS-CoV-2 sequence repositories.

***Design and testing of new SARS-CoV-2 E forward primers.*** Newly designed *E*-based primers (named *E-OM-1*, *E-OM-2*, *E-WuOM-1*, and *E-WuOM-2*) (Table 1) covered the same region as the assay’s *E* forward primer. They were assessed for the presence of hairpins, primer-primer interactions, and their melting temperatures using OligoAnalyzer (Integrated DNA Technologies). In addition, *E-OM-1*, *E-OM-2*, *E-WuOM-1*, and *E-WuOM-2* were analyzed for inclusivity and overall PCR efficiency using the same GISAID set of SARS-CoV-2 sequences as described above and in the DECIPHER package.

**Table 1.** *Primer sequences and predicted overall PCR efficiencies of E primers.* Sequence, melting temperature (Tm), and predicted PCR efficiency for either the Delta or Omicron variants for *E* primers *(E-OM-1, E-OM-2, E-WuOM-1, and E-WuOM-2)* were used in this study. Overall PCR efficiencies were calculated based on a perfectly matched template. Bold text indicates the C-A mismatch for the *E* primer (original) and substituted bases for other designed *E* primers.

Forward Primer	Sequence (5’ to 3’)	Calculated Tm	Predicted PCR efficiency	
			Delta	Omicron
*E*(original)	TTCGGAAGAGA**C**AGGTACGTT	62.5°C	100%	1%
*E-OM-1*	CGTTTCGGAAGAGA**T**AGGTACGTTA	63.2°C	26%	140%
*E-OM-2*	CGTTTCGGAAGAGA**T**AGGTACGTT	63.5°C	26%	140%
*E-WuOM-1*	CGTTTCGGAAGAGA**Y**AGGTACGTTA	T/C 63.2/65.2°C	477%	140%
*E-WuOM-2*	CGTTTCGGAAGAGA**Y**AGGTACGTT	T/C 63.5/65.6°C	477%	140%

The *E-OM-1*, *E-OM-2*, *E-WuOM-1*, and *E-WuOM-2* primers were paired with the original *E* reverse primer for RT-qPCR testing using serial dilutions of the synthetic RNA templates described earlier.

**Calculations of amplification efficiency and statistical analysis.** Cq values and corrected individual amplification efficiencies were determined from the amplification curve [13] using the web-based LinRegPCR [14]. Only Cq values lower than 34.0 were used in the analysis. One-way or two-way ANOVA with a Tukey post-hoc test in R was used to determine statistical significance (*p* < 0.001) when comparing delays in Cq values and amplification efficiency using synthetic RNA templates.

## Results

In November 2021, Pango Lineage B.1.1.529/BA.1 (Nexstrain, 21K WHO’s technical Advisory Group, Omicron) emerged in South Africa and rapidly developed into a public health emergency. The Omicron lineage, as referred to hereafter, differs from its predecessor Delta in severity and acquisition of important genetic adaptations. Some of these mutations favoured host cell entry and dampened neutralizing antibody activity among vaccinated and previously infected individuals [15-19]. As a result, only a handful of therapeutic interventions using monoclonal antibody cocktails are effective against Omicron. Thus, rapid identification of the viral genetic variant is of utmost clinical relevance [15-18].

***Sequence surveillance of SARS-CoV-2 variants.*** We downloaded, aligned, and analyzed up to ∼10,000 published, high-quality sequences for VOCs Alpha (B.1.1.7), Beta (B.1.351), Gamma (P.1), Delta (B.1.617), Omicron (B.1.1.529/BA.*) and subvariants (BA.1, BA.1.1, BA.2, BA.2.12.1, BA.2.3.20, BA.2.75, BA.3, BA.4, BA.4.6, BA.5, BF.7, BJ.1, BQ.1, BQ.1.1, XBB), and VOIs Lambda (C.37) and Mu (B.1.621) for mismatches to our assay’s primers (Table 2(a,b)). Mismatches, excluding mixed bases, that were predicted to reduce PCR efficiency were flagged as likely to have a significant impact on assay performance. We define PCR efficiency as the overall efficiency of the PCR reaction, which includes (*i*) the efficiency of hybridization represented by the stability of the primer-template duplex, (*ii*) the efficiency of elongation as it relates to the ability of the polymerase to extend off the 3’ end, and (*iii*) the efficiency of the exponential increase in fluorescence values during amplification. Consequently, we defined the criteria to further investigate a particular mismatch if it (*i*) was predicted to cause a reduction to less than 10% in overall PCR efficiency as compared to the perfectly matched template, and (*ii*) was found in more than 10% of published sequences for a particular variant, or (*iii*) more than 5% of published sequences were predicted to contain primer mismatches in at least two primer target regions.

**Table 2.** *Sequence surveillance and predicted overall PCR efficiency of SARS-CoV-2 variants.* SARS-CoV-2 (**A**) and Omicron subvariants (**B**) sequences were downloaded from GISAID, aligned, and analyzed for mismatches to assay primers using the predicted PCR efficiency tool in DECIPHER. Columns summarize the number of sequences included in the analysis for each SARS-CoV-2 variant along with the percentage of these sequences that contained mismatches predicted to decrease PCR efficiency to less than 10% of a perfectly matched template. The last column provides the percentage of sequences with primer-template mismatches that are predicted to impact assay performance that are in two or more target regions.

**A.**								
Variant	Sequences analyzed	*Nf* (%)	*Nr* (%)	*Ef* (%)	*Er* (%)	*Sf* (%)	*Sr* (%)	2 + regions (%)
Wuhan	9,075	0.26	1.12	0.06	0.04	0.12	0.65	0.01
Alpha	8,829	0.17	0.03	0.78	0.16	0.09	1.33	0.00
Beta	4,904	0.10	0.02	0.00	0.16	0.22	0.10	0.00
Gamma	9,482	0.13	0.33	0.12	0.01	0.69	0.12	0.00
Delta	9,365	0.15	0.19	0.04	0.00	0.04	0.07	0.00
Lambda	8,172	0.83	**28.01**	0.27	0.00	2.14	0.45	0.32
Mu	5,427	0.33	0.79	0.09	0.06	0.35	0.15	0.02
Omicron	6,438	0.12	0.14	**99**.**44**	0.02	0.00	0.03	0.28

**B.**								
Omicron Subvariant	Sequences analyzed	*Nf* (%)	*Nr* (%)	*Ef* (%)	*Er* (%)	*Sf* (%)	*Sr* (%)	2 + regions (%)
BA.1	3,132	0.00	0.10	**98.24**	0.03	0.00	0.10	0.19
BA.1.1	1,807	0.06	0.28	**96**.**57**	0.00	0.55	0.66	1.44
BA.2	9,266	0.12	0.09	**99**.**96**	0.00	0.01	0.00	0.22
BA.2.12.1	1,958	0.20	0.10	**99**.**95**	0.00	0.00	0.05	0.36
BA.2.3.20	789	0.13	0.00	**100**.**0**	0.00	0.38	0.00	0.51
BA.2.75	1,700	0.24	0.88	**99**.**94**	0.06	0.06	0.00	1.18
BA.3	170	0.00	0.00	**90**.**00**	0.00	0.00	0.00	0.00
BA.4	9,208	0.08	0.11	**99**.**91**	0.02	0.02	0.08	0.27
BA.4.6	364	0.00	0.55	**99**.**73**	0.00	0.00	0.55	1.10
BA.5	8,811	0.47	0.30	**99**.**97**	0.05	0.37	0.14	1.27
BF.7	1,611	0.63	0.06	**99**.**87**	0.00	1.19	0.00	1.88
BJ.1	118	0.00	0.00	**100**.**0**	0.00	0.00	0.00	0.00
BQ.1	3,530	0.99	3.60	**99**.**94**	0.00	0.14	0.14	**4**.**87**
BQ.1.1	3,571	0.03	0.22	**100**.**0**	0.00	0.17	0.08	0.50
XBB	1,627	0.06	0.00	**100**.**0**	0.00	0.18	0.18	0.43

No assay primer mismatches were identified in VOCs Alpha, Beta, Gamma, and Delta or VOI Mu that satisfied the aforementioned criteria. Conversely, VOI Lambda exhibited one mismatch in the annealing region of the *N* reverse (*Nr*) primer that was predicted to reduce PCR efficiency for the *N* region to 2.12% and was identified in 28.01% of the 8,172 analyzed sequences (Table 2A). Further analysis confirmed no other significant mismatches existed under other primer annealing regions in the Lambda sequences. Because of this, and due to the lack of global spread of this variant, analysis of the mutation in Lambda was not pursued further.

**Figure 1.**
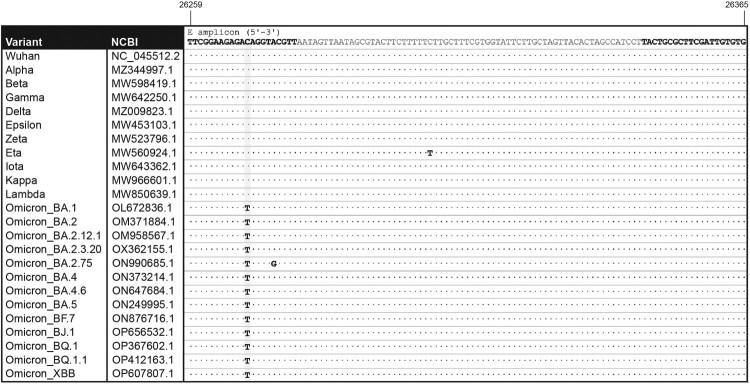
*Alignment of the E primer pair to representative target sequences.* Location of forward and reverse *E* primers within the *E* gene are indicated (bold) in the sequence of SARS-CoV-2 (Wuhan strain, NC_045512.2). The amplicon sequences are shown for the following SARS-CoV-2 variants: Alpha (MZ344997.1), Beta (MW598419.1), Gamma (MW642250.1), Delta (MZ009823.1), Epsilon (MW453103.1), Zeta (MW523796.1), Eta (MW560924.1), Iota (MW643362.1), Kappa (MW966601.1), Lambda (MW850639.1), Omicron BA.1 (OL672836.1), BA.2 (OM371884.1), BA.2.12.1 (OM958567.1), BA.2.3.20 (OX362155.1), BA.2.75 (ON990685.1), BA.4 (ON373214.1), BA.4.6 (ON647684.1), BA.5 (ON249995.1), BF.7 (ON876716.1), BJ.1 (OP656532.1), BQ.1 (OP367602.1), BQ.1.1 (OP412163.1) and XBB (OP607807.1).

VOC Omicron showed a single C-A mismatch, 10 bases upstream of the 3’ end of the *E* forward (*Ef*) primer (Figure 1). This mutation under *Ef* was predicted to reduce overall PCR efficiency for the *E* region to 1.22% as compared to a perfectly matched template. This mismatch was identified in 99.4% of 6,438 analyzed Omicron sequences; however, no other significant mismatches were identified among the *N* and *S* primer annealing regions. The *Ef* C-A mismatch was present in 98.24% of BA.1, 96.57% of BA.1.1, 99.96% of BA.2, 90.0% BA.3, 99.91% BA.4, 99.97% BA.5, 99.87% BF.7, 99.94% of BQ.1, 99.94% of BA.2.75, 99.73% of BA.4.6, and 100% of BA.2.3.20, BQ.1.1, BJ.1 and XBB sequences indicating that the mutation was prevalent in sub-lineages of Omicron (Table 2). Interestingly, a new Omicron subvariant, BA.2.75, exhibited an additional mismatch in *Ef*, 5 bases upstream of the 3’ end (26275A > C) and, whereas amplification was not compromised, the Cq delay in this scenario increased [CqΔ(*E*-*N*): 10.71 ± 3.62; CqΔ(*S*-*N*): −0.33 ± 0.56; RPP30: 31.48 ± 3.13; n = 20]. The specificity of the C-A mismatch to Omicron variants is relevant for differentiating them from the highly mutated Delta variant in a single amplification reaction. Accordingly, whereas mismatches raise concern when considering the overall performance of a molecular assays, a viral variant with an imperfect primer-template annealing and no alteration in amplification efficiency might have a unique molecular screening signature value for variant assessment. This scenario is most likely to occur when redundancy is built into the assay due to the amplification of multiple target regions.

**Figure 2.**
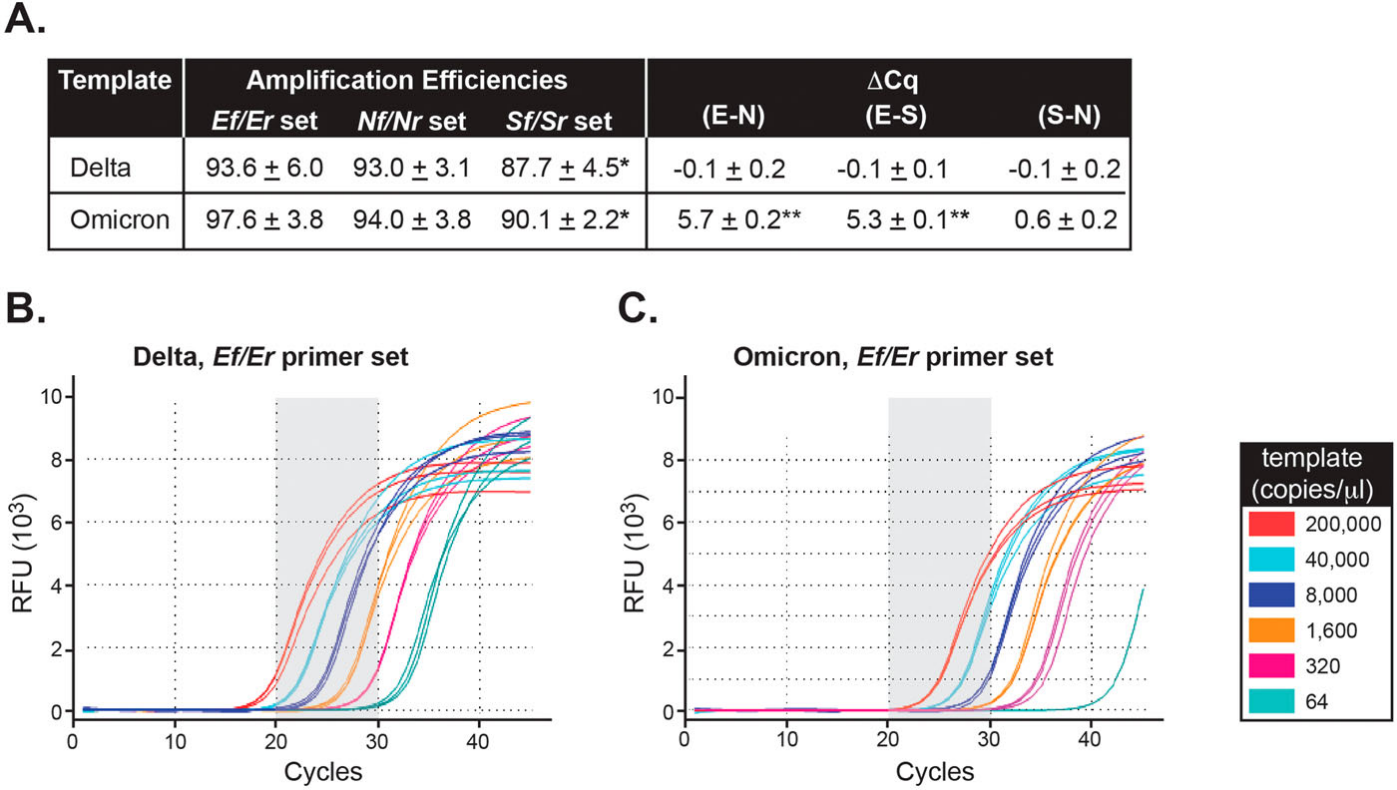
*The Ef mismatch causes a quantification cycle delay but does not affect amplification efficiency in Omicron.* **A**, Average amplification efficiencies and average ΔCq values for each target region were determined based on the slope of the individual amplification curves using Delta or Omicron as template and each of the *Nf/Nr*, *Ef/Er*, and *Sf/Sr* set of primers. *Statistically significant difference in amplification efficiency between the *S* set of primers and either *E* or *N* (*p* < 0.001). **Statistically significant difference for ΔCq values between Delta and Omicron (*p* < 0.001). (**B-C**) Background-adjusted fluorescence curves generated using the CFX Maestro Software (BioRad) obtained when using the *Ef* primer and serial dilutions of Delta (**B**) and Omicron (**C**) synthetic templates (at 200,000, 40,000, 8,000, 1,600, 320, or 64 copies/μl). All samples were run in triplicate.

***Impact of Ef primer mismatch on Delta and Omicron detection in vitro.*** Serial dilutions of synthetic Delta and Omicron RNA templates were processed using our in-house SARS-CoV-2 assay and overall PCR efficiencies, defined as the slope of the individual amplification curves [20-22], were determined for the three assay target regions (*N*, *E*, and *S* genes) (Figure 2 and Sup. Figure 1A). Amplification efficiencies were 93.0 ± 3.1 and 94.0 ± 3.8 for *N*, 93.6 ± 6.0 and 97.6 ± 3.8 for *E*, and 87.7 ± 4.5 and 90.1 ± 2.2 for *S* using Delta and Omicron templates, respectively (Figure 2(a)). The amplification efficiency for the *S* gene was lower as compared to that of *E* and *N* but this difference was relatively small and independent of the template use in the reaction (Figure 2(a), *p* < 0.001).

Consequently, we evaluated Cq values among *N*, *E*, and *S* genes using either Delta or Omicron as the template. When the reaction was carried out using Delta as template, ΔCqs were small between *E* vs. *N* (−0.1 ± 0.2) and *E* vs. *S* (−0.1 ± 0.1) despite lower *S* amplification efficiency (Figure 2(a)). Interestingly, when Omicron was the template in the reaction, the average Cq delay for amplification of the *E* region was 5.7 ± 0.2 and 5.3 ± 0.3 cycles compared to the *N* and *S* regions, respectively (Figure 2(a), Sup. Figures 2A-B, Sup. Figures 1B-E). In comparison, the ΔCq between *S* and *N* was 0.6 ± 0.2 for the same template. The ΔCq for *E-N* and *E-S* versus *S-N* were statistically significant (*p* < 0.001).

In summary, Cq values and amplification efficiencies were comparable among the *N*, *E*, and *S* genes when reactions were carried out using the Delta template for which the *E* primer showed no mismatches. Remarkably, a Cq delay, but no change in amplification efficiency, was exclusively identified for the *E* gene using the Omicron template. This suggests that the C-A mismatch within *Ef* likely impacts the first few rounds of amplification due to mispriming, after which, newly synthesized templates perfectly match the *Ef* primer and thus, amplification is no longer impacted. The *in vitro* results support the *in-silico* predictions and indicate that a Cq delay for *E* amplification is a unique feature of the Omicron variant.

**Figure 3.**
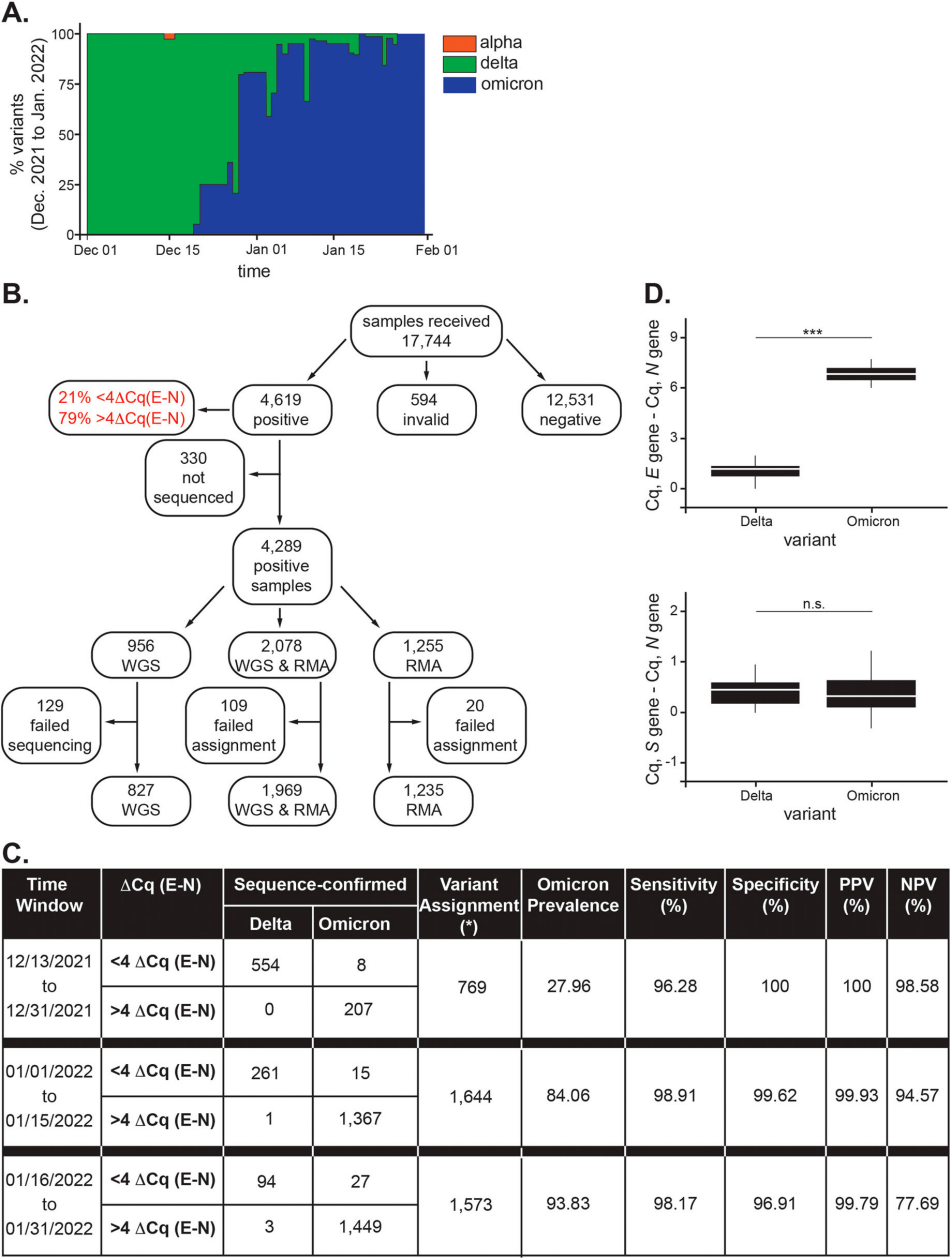
*Screening of SARS-CoV-2-positive clinical samples for Omicron by RT-qPCR and sequencing.* **A**, Summary of weekly distribution of SARS-CoV-2 variants circulating in Southwest Virginia between December 1, 2021 and January 31, 2022 as determined by RT-qPCR, RMA, and WGS sequencing. **B**, From December 13, 2021 to January 31, 2022, samples (17,744) were received and analyzed for SARS-CoV-2 presence using our FDA-authorized SARS-CoV-2 developed test [7]. Of these samples, there were 4,619 positives (defined as those that generate a positive signal in at least 2 of the SARS-CoV-2 genes and the housekeeping *RPP30* gene). Samples were considered invalid (594) when there was no detectable expression of the *RPP30* gene. Among positives, 330 samples were deemed not suited for sequencing due to their RNA quality or high Cq value for SARS-CoV-2 genes. Of the remaining 4,289 positive samples, 956 were subjected to whole genome sequencing, 1,255 to rapid mutational analysis, and 2,078 to both sequencing methods. A few samples failed sequence analysis (indicated in **B**) or were not assigned a variant type by Pangolin. **C**, Summary shows the number of samples confirmed by sequencing and assigned to a specific variant. * Indicates samples for which RMA and WGS provided opposite variant assignments [22 unassigned samples for >4 ΔCq (*E-N*) and 23 unassigned samples for <4 ΔCq (*E-N*)]. PPV and NPV indicate positive and negative predicted values, respectively. **D.** Box plots represent mean differences between *E*, or *S*, and *N* Cq values for positive samples tested (n = 20 for each Delta and Omicron). Error bars represent SD (*p* < 0.001).

***Screening of clinical specimens for Omicron using the Cq delay.*** The Southwest region of Virginia, where this study was carried out, experienced a rise in the Delta wave in the summer of 2021, with its first case being reported by our laboratory on June 28, 2021. With weekly increases of ∼18%, Delta was established as the dominant variant by August 2021 with 100% of positive samples confirmed by either rapid mutational analysis (RMA) or whole genome sequencing (WGS) (data not shown). By mid-December 2021, Delta remained the dominant VOC, though a few positive Alpha samples (0.86%) was detected on December 16, 2021 (Figure 3(a)). On December 21, 2021, our laboratory reported the first case of the B.1.1.519/BA.1 Omicron variant, confirmed by WGS, in the region. Omicron rapidly replaced Delta as the most prominent VOC in the region from that day forward (Figure 3(a)).

A total of 17,744 nasopharyngeal samples, collected between December 13, 2021 and January 31, 2022, were analyzed by our laboratory for SARS-CoV-2. Of this pool, 4,619 were reported positive, as defined by the amplification in at least two SARS-CoV-2 genes and the detection of the human housekeeping *RPP30* gene [Figure 3(b), [7]]. Furthermore, 12,531 and 594 samples were negative or invalid, respectively, based on the absence of amplification for any SARS-CoV-2 or *RPP30* genes (Figure 3(b)).

Positive samples were analyzed by RMA, which involved sequencing four-to-six PCR amplicons spanning known regions of SARS-CoV-2 rich in Omicron mutations (between 15 and 20 mutations depending on the Omicron subvariant), as well as by WGS. Among 4,619 positives (∼79% showed a > 4Cq), 330 samples were not sequenced due to poor quality or sample redundancy. The remaining 4,289 positive samples were characterized by RMA (1,255 samples), WGS (956 samples), or both (2,078 samples) (Figure 3(b)). A few sequences failed assignment to a specific variant or were not included due to low template quality or quantity. Thus, of the 4,031 positive samples sequenced, 3,986 were unambiguously characterized at the genomic level and the sensitivity of the assay was validated at three different intervals of time for which the prevalence of Omicron was different. Accordingly, sensitivity was 96.28% (12/13/2021–12/31/2021, Omicron prevalence 27.96%), 98.91% (01/01/2022–01/15/2022, Omicron prevalence 84.06%), and 98.17% (01/16/2022–01/31/2022, Omicron prevalence 93.83%) for those samples that exhibited a ΔCq (*E-N*) > 4 and were sequence-confirmed as Omicron. Specificities of 100%, 99.62%, and 96.91%, PPV values of 100%, 99.93%, and 99.79%, as well as NPVs of 98.58%, 94.57%, and 77.69% were determined for each of the three consecutive intervals, respectively (Figure 3(c)). The performance of our assay was independently validated by the Department of General Services, Division of Consolidated Laboratory Services (DCLS), VA, U.S. using the assay primers reported in Sup. Table 1. Results from DCLS’ analyses show that samples exhibit a comparable Cq delay exclusively for those specimens confirmed as Omicron (ΔCq (*E-N*): 6.70 ± 1.28, ΔCq (*S-N*): 0.42 ± 0.46, n = 20) but not Delta (ΔCq (*E-N*): 1.07 ± 0.70, ΔCq (*S-N*): 0.53 ± 0.71, n = 20) by whole genome sequencing (Figure 3(d)).

**Figure 4.**
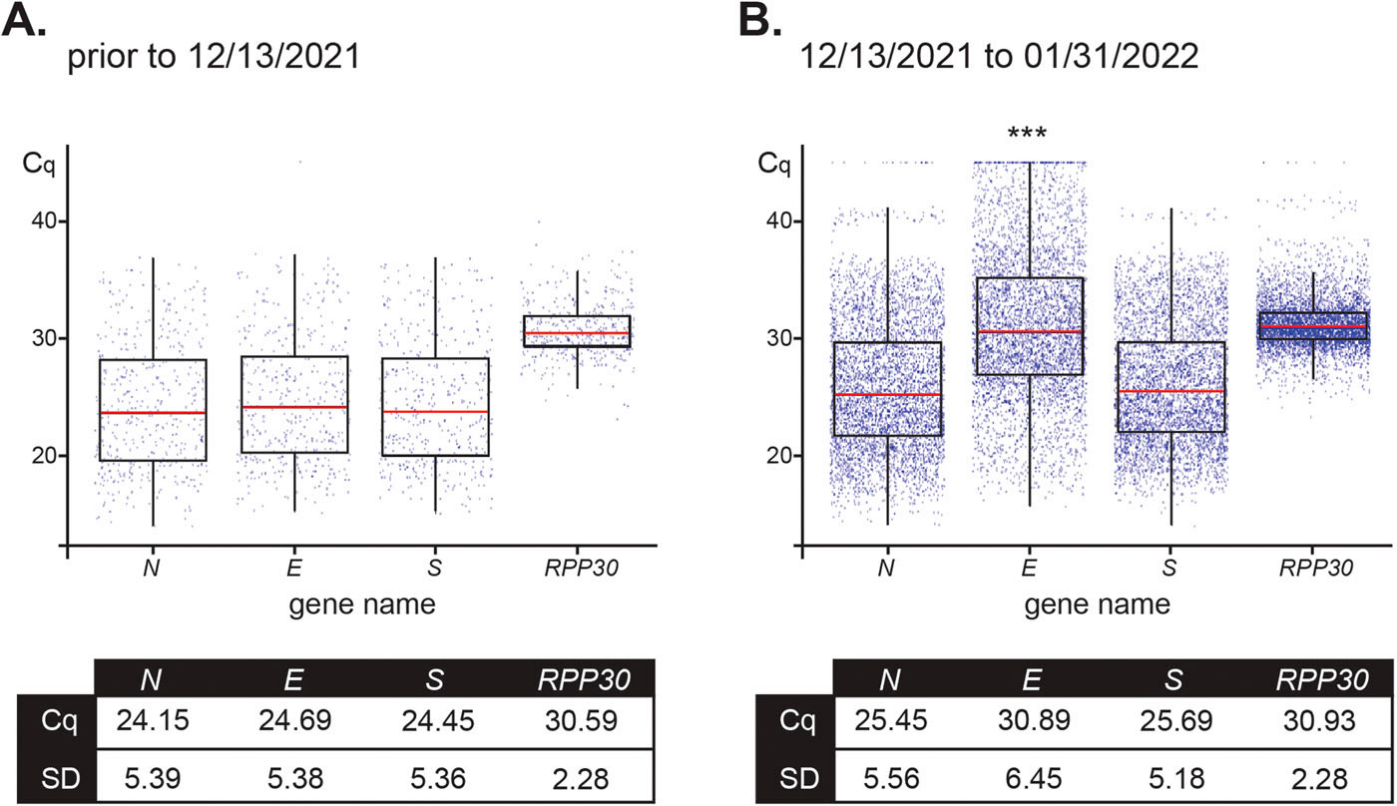
*Distribution of Cq values for assay primers show a variant-dependent delay for amplification of E.* Box plots represent mean Cq ± SD values for the SARS-CoV-2 genes *N*, *E*, and *S*, and housekeeping *RPP30* for 436 positive samples received prior to December 13, 2021 (100% Delta as determined by RMA and/or WGS) (**A**) and 4,619 positive samples analyzed from December 13, 2021 to January 31,2022 (79% Omicron and 21% Delta as determined by RMA and/or WGS) (**B**).*** Indicates significant difference between the *E* value prior to 12/13/2021 and from 12/13/2021–01/31/2022 as determined by two-way ANOVA followed by a Tukey test (*p* < 0.0001).

For the 4,619 positive samples collected between 12/13/2021 and 01/31/2022, overall Cq values were: 25.45 ± 5.56 for *N*, 30.89 ± 6.45 for *E*, 25.69 ± 5.18 for *S*, and 30.93 ± 2.28 for *RPP30* (Figure 4, right panel). These results were compared to the 436 positive nasopharyngeal samples collected prior to 12/13/2021 and that were confirmed as a Delta variant by WGS. Results for Delta samples were: 24.15 ± 5.39 for *N*, 24.69 ± 5.38 for *E*, 24.45 ± 5.36 for *S*, and 30.59 ± 2.28 for *RPP30* (Figure 4, left panel). Importantly, differences in *RPP30* values, the internal control, between samples collected before and after the beginning of the Omicron wave (12/13/2021), as well as among positive-sequenced samples of the two variants compared within the time window of our study (12/13/2021–01/31/2022), proved to be statistically non-significant. As only one copy of the *N*, *E*, and *S* genes are present in the genome of SARS-CoV-2, their corresponding Cq values after amplification are expected to be comparable if primer-template exhibits perfect annealing and comparable efficiencies as was the case for Delta, but not Omicron (Figure 4). The predicted mismatch in *Ef* resulted in a distinct delay in *E* Cq as compared to the Cq values for Omicron *N* and *S* genes.

***Evaluation of new Ef primer designs to broadly detect SARS-CoV-2 variants.*** A molecular signature of the *E* primer containing the C-A mismatch is that its melting temperature (Tm) dropped by 2.6°C compared to the corresponding matched primer. Accordingly, we evaluated the possibility that *E* primers designed to compensate for the Tm drop might detect all known variants.

To address this hypothesis, we designed and tested four *E* forward primers (named *E-OM-1*, *E-OM-2*, *E-WuOM-1*, and *E-WuOM-2*) (Table 1) whose sequences were extended 3 bases (CGT) at the 5’ end (E-OM-2 and *E-WuOM-2*) or 3 bases (CGT) at the 5’ and 1 base (A) at the 3’ end (*E-OM-1* and *E-WuOM-1*), compared to the original *E* forward primer. Addition of these nucleotides increased their calculated melting temperatures within two degrees of the Tm of the original reverse primer (Table 1). *E-OM-1* and *E-OM-2* had the “C” located 10 bases upstream of the 3’ end replaced by “T” and, thus, the primers were predicted to perfectly anneal Omicron variants. In contrast, *E-WuOM-1* and *E-WuOM-2* had the “C” replaced by “Y” (mix of pyrimidines, C or T) and, thus, could perfectly match Omicron and all other variants (Table 1).

These new primers were analyzed for their predicted overall PCR efficiency using perfectly matched templates. All four primers were predicted to detect either Delta or Omicron templates with at least 25% efficiency relative to the original *Ef* for Delta (Table 1). *E-OM-1*, *E-OM-2*, *E-WuOM-1*, and *E-WuOM-2* were analyzed for their predicted inclusivity in 69,620 aligned SARS-CoV-2 genomes used in Table 2A. Less than 0.33% of these published sequences contained mismatches that were predicted to impact our assay’s performance if *Ef* were replaced by any of the newly designed *E* forward primers [for *E-OM-1*, 229 mismatched sequences (0.33%); *E-OM-2*, 228 mismatched sequences (0.33%); *E-WuOM-1*, 28 mismatched sequences (0.04%); *E-WuOM-2*, 27 mismatched sequences (0.04%)].

**Figure 5.**
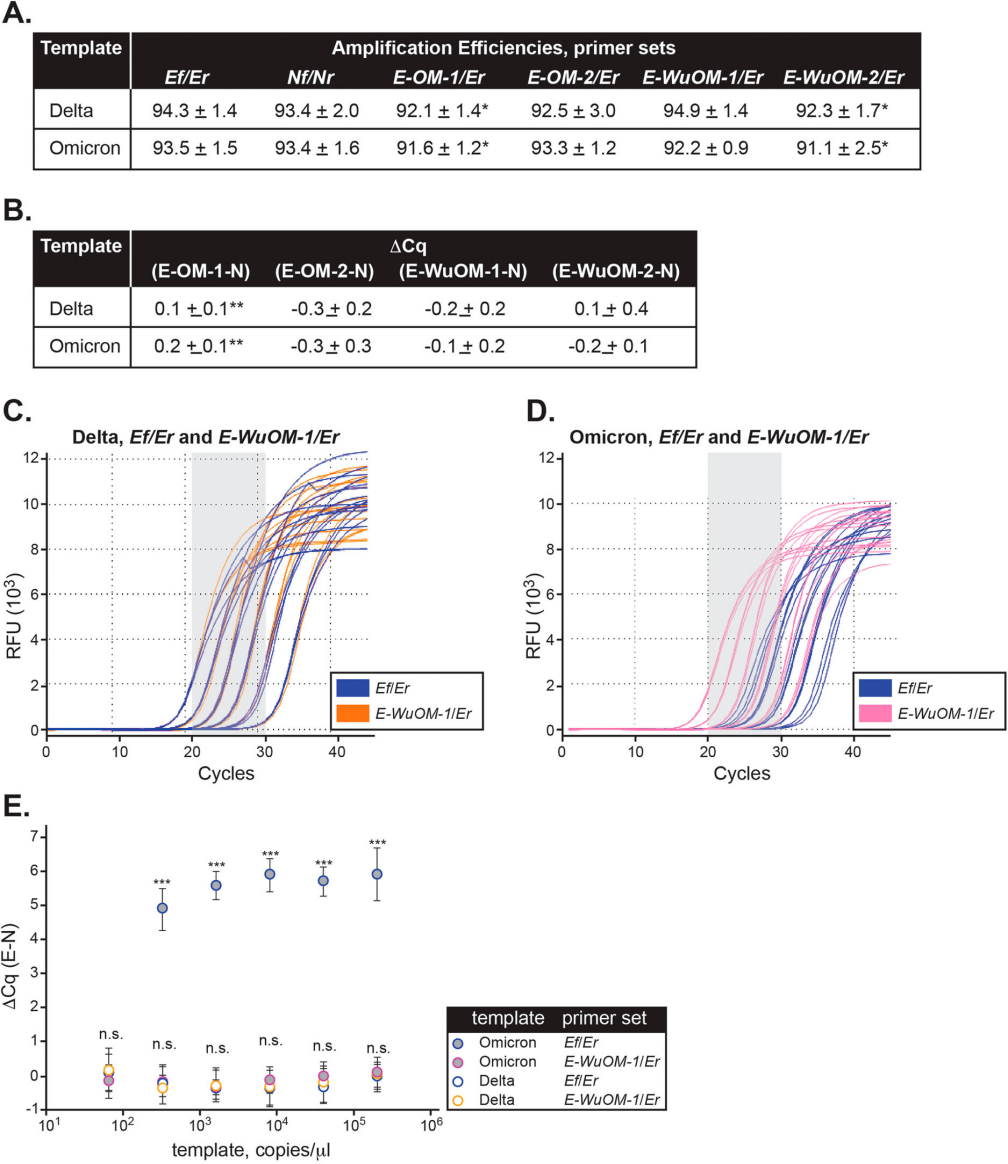
*Newly designed E forward primers efficiently recognize Delta and Omicron variants. Ef, E-OM-1*, *E-OM-2*, *E-WuOM-1*, or *E-WuOM-2* primers were paired with *Er* (Table 1 and Sup. Table 1) and *Nf* paired to *Nr* for RT-qPCR using Delta or Omicron synthetic templates. Decreasing one-fifth dilutions series of each template was used in each assay and amplification efficiencies (**A**) and ΔCq values (**B**) calculated as described in Materials and Methods. * Indicates statistically significant difference between the amplification efficiency of *E-OM-1*/*Er*, or *E-WuOM-2*/*Er* versus *Ef*/*Er* (*p* < 0.001). ** Indicates a statistically significant difference between the Cq delay generated by *E-OM-1* versus *E-OM-2*, *E-WuOM-1*, or *E-WuOM-2* primers (*p* < 0.001). A typical run using the *Ef*/*Er* and *E-WuOM-1*/*Er* primer sets for Delta and Omicron is depicted in **C** and **D**, respectively. Serial dilutions of each template show amplification curves matched for both primer sets using Delta as a template (blue and orange lines in **c**) and shifted back using Omicron for the new *E-WuOM-1*/*Er* set (pink, **D**). **E**. Average ΔCq calculated for each concentration of the four primer sets used to generate the plots depicted in **C** and **D**. Standard deviation of the mean is shown in each case. ***Indicates amplification using the *Ef*/*Er* primer set and Omicron as template is significantly different (*p* < 0.001) compared to *WuOM-1* or *Ef*/*Er* and Delta template or *WuOm-1*/*Er* and Omicron as a template. No statistical difference (n.s.) was observed among *WuOM-1* or *Ef*/*Er* and Delta template or *WuOm-1*/*Er* and Omicron as a template.

*N, Ef, E-OM-1*, *E-OM-2*, *E-WuOM-1*, and *E-WuOM-2* primers were tested in separate amplification reactions using a dilution series of the synthetic Omicron or Delta RNA templates (Figure 5). Delta was chosen to represent all SARS-CoV-2 variants that perfectly matched the *Ef* primer. We observed (*i*) the amplification efficiencies and Cq values for *E-OM-1*, *E-OM-2*, *E-WuOM-1*, and *E-WuOM-2* were comparable to the values calculated for the *N* gene regardless of the template used (Figure 5(a,b)), (*ii*) there was no statistical significance in ΔCq, *i.e.* (*E* –*N*), using *E-OM-2*, *E-WuOM-1*, or *E-WuOM-2* and either template (Figure 5(b)), (*iii*) *E-OM-1*, *E-OM-2*, *E-WuOM-1*, and *E-WuOM-2* were able to detect Omicron by an order of magnitude greater than the original *Ef* assay primer (Figure 5(c,d), Sup. Figure 2), and (*iv*) *E-OM-1*, *E-OM-2*, *E-WuOM-1*, and *E-WuOM-2* were able to detect either Omicron or Delta without a significant delay in Cq as compared to *Nf*/*Nr* (Figure 5(e)). Therefore, *E-OM-1*, *E-OM-2*, *E-WuOM-1*, or *E-WuOM-2* could be successfully used, instead of *Ef*, to detect templates containing, or not containing, the C-A mismatch without impacting amplification efficiency and without causing a significant delay in the Cq value for the *E* gene (Sup. Figure 2).

**Figure 6.**
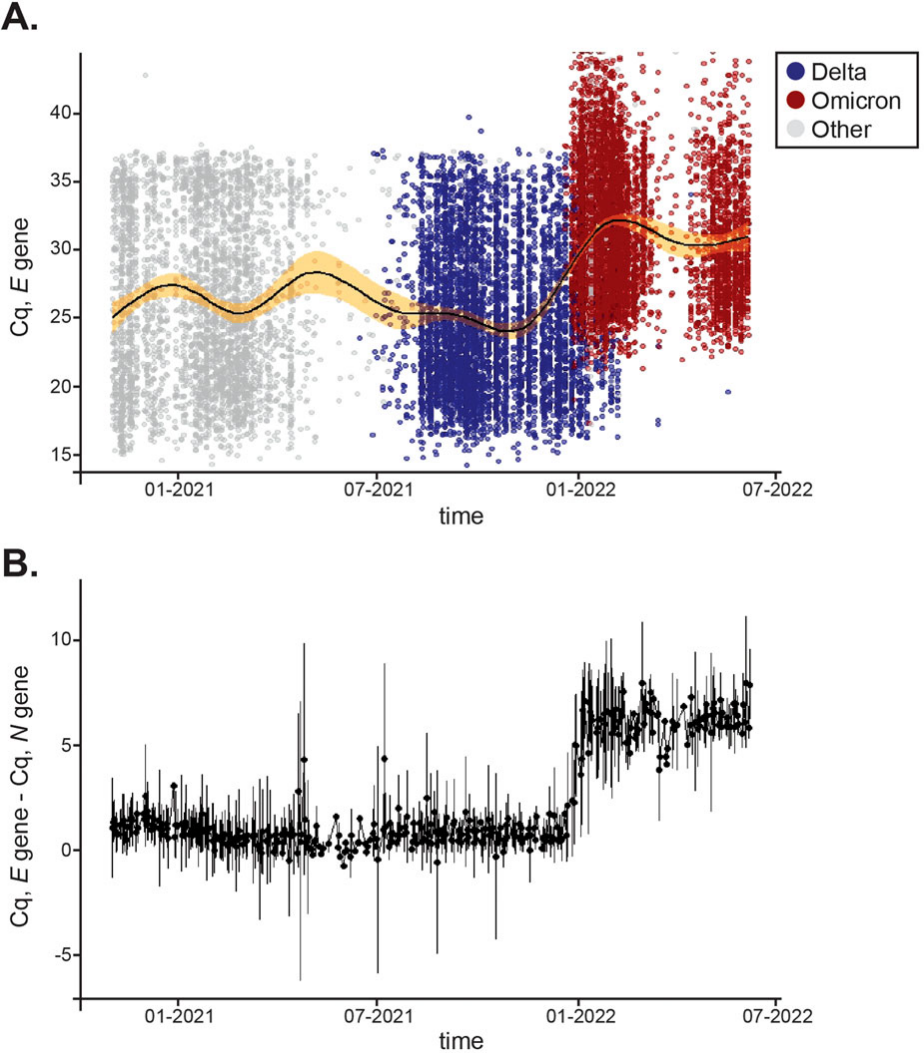
*Distribution of Cq values for the E gene across variants over time.* **A**, 16,351 positives samples were reported between January 1,2021 and July 7, 2022. Distribution of Cq values for the *E* gene are shown for Delta (blue) and Omicron (red) over time. Other variants’ *E* Cq values are represented by a gray circle. A solid black line displays average Cq for *E* and the shaded area shows 99% confidence intervals. **B**, Mean differences between *E* and *N* Cq values for positive samples tested within the same time frame. Error bars represent SD.

***Analytical comparison of Cq values over time showed that delays existed only for the E gene in clinical samples.*** We then asked whether there were other instances during the pandemic where Cq delays would have existed for the *N*, *E*, and *S* genes despite the fact that no mismatches were identified in our customized primers. Virginia experienced various waves of COVID-19 infections driven by emerging VOCs. Between January 1, 2021 to July 7, 2022, our laboratory analyzed 148,572 clinical samples and reported 16,351 positives that included Alpha, Beta, Delta, Epsilon, Gamma, Iota, and Omicron variants in addition to several VOIs reported by WHO. For simplicity, and consistency during the analysis, variants other than Delta and Omicron are depicted in gray in Figure 6 and Sup. Figure 3. All positive samples collected throughout that period were plotted for their average Cq value for *E* (Figure 6(a)), *N*, and *S* (Sup. Figure 3A). To account for differences in viral load, we plotted the difference between Cq values for the *N* and *E* genes for which only one copy of each exists in the SARS-CoV-2 genome. Figure 6(b) shows that the ΔCq (*E* – *N*) difference was negligible pre-Omicron and that Cq delays solely persisted during Omicron infection. In agreement, Cq values for *N* and *S* remained steady throughout the time frame analyzed (Sup. Figure 3C). Thus, we demonstrate that a signature molecular shift that solely results from amplification of the Omicron variant can be used in the field for public health surveillance and as a surrogate marker in the clinic.

## Discussion

The uninterrupted emergence of SARS-CoV-2 variants poses a challenge to molecular diagnostic tests that rely on RT-qPCR for virus detection. Built-in redundancy in gene amplification ensures that molecular-based assays are capable of providing a positive identification of SARS-CoV-2 variants even when new mutations lead to gene amplification dropouts. In fact, an increasing number of mutations and deletions in the SARS-CoV-2 genome affect the analytical sensitivity and efficiency of primers and probes used for amplification in routine assays [23]. For example, the Alpha variant B.1.1.7, initially discovered in the United Kingdom, displays a remarkably large number of single nucleotide variations and deletions in the *ORF1a*, *ORF1b*, *S*, *ORF8*, and *N* genes, some of which facilitate virus transmission [1]. The Alpha *S* gene, in particular, contains a deletion, del69/70, in the spike protein that results in *S* gene target failure (SGTF) during PCR amplification. Initially, failure to detect the *S* gene by most widely used commercial diagnostic assays was alarming; however, as other viral genes were identified and SGTF was confirmed by whole genome sequencing to correlate with Alpha variants, *S* dropout became a reliable marker to rapidly assess the prevalence of B.1.1.7 in the population [2,9]. Other mutations present in Alpha (*e.g.* T478K, E484A, and N501Y in the spike protein) were also used in laboratory developed tests for rapid variant identification [10]. However, what, *a priori*, was a valuable epidemiological tool became outmoded when emerging new variants, including Delta and Omicron BA.1, but not the BA.2 sub-lineage, displayed the presence of an *S* gene deletion and SGTF after amplification, a result that precluded effective identification among circulating strains. Furthermore, as genomic alterations among new variants increased, and primer binding and PCR amplification efficiency became compromised, positive samples containing a low copy number of viral particles could fail amplification and, consequently, be reported as false-negatives. Thus, a specific amplification test, like ours, that does not compromise sensitivity and easily distinguishes between Omicron variants and other circulating lineages turned into a desirable laboratory tool. Importantly, our findings arrived at a time when early reports acknowledged that Omicron escaped neutralization antibodies and that the existing vaccines provided limited protection against the new variant [24,25]. It remains to be seen whether the Cq delay holds its promise as a molecular surrogate marker among emerging variants or whether, as was the case for the *S* gene dropout characteristic of the Alpha variant, it will only be appropriate for variant-specific analysis. Of note, and despite not being the subject of this paper, our most recent analysis showed that nearly 5% of BQ.1 published sequences contain a mismatch within one of the *N* primers and thus, continuous monitoring of our assay’s performance is warranted. Therefore, genomic surveillance and testing of mismatches should serve as a blueprint for the development and monitoring of an assay’s performance of molecular diagnostic tests.

By mid-December 2021, hospital news was distressing, as monoclonal antibody cocktails, once successful for treatment of severe COVID-19 in high-risk people infected with SARS-CoV-2, were rendered “powerless” against Omicron [15]. Early findings showed that only two monoclonal antibodies directed towards the spike protein, sotrovimab and DXP-604 (not authorized in the U.S.), retained some ability to neutralize Omicron [16,18,26]. As a result, health officials in the U.S. were prompted to consider prioritizing the distribution of sotrovimab to areas with high prevalence of Omicron and large numbers of hospitalizations, at least until production of sotrovimab increased and/or antiviral drugs effective against Omicron were approved (Paxlovid, Pfizer and Molnupiravir, Merck were not yet authorized by the FDA in December, 2021). It was imperative, then, to implement a strategy to differentiate individuals infected with Delta, and thus receptive to standard-of-care monoclonal therapy, from those with Omicron, for which only a specific neutralizing antibody was effective.

Whole genome sequencing (WGS) provides a comprehensive picture of genomic alterations in SARS-CoV-2 that facilitates lineage assignment. Although of epidemiological importance, WGS faces practical challenges that makes its implementation as a rapid diagnostic tool unrealistic. As such, we first developed a cost-effective, “rapid mutational analysis (RMA)” strategy that relies on Sanger sequencing of amplicons containing hallmark mutations. However, as mutational information of the many variants exists but only a few regions of the genome are sequenced, only a rapid, yet clinically relevant, lineage call can be made (e.g. Delta *vs.* Omicron). Alternatively, data analyses showed that a significant Cq delay enabled us to differentiate Omicron from Delta variants in a single amplification reaction, thus, providing an accurate, affordable, and scalable test of clinical value. With this tool in hand, we were able to provide local clinicians with an early assessment of the patient’s sample variant for better allocation of monoclonal antibodies resources.

Lastly, it seems unrealistic to implement broad surveillance using WGS while laboratories struggle to perform the massive number of PCR tests that this pandemic is causing them to run. A more targeted approach for WGS surveillance and a broad implementation of variant-specific PCR detection could be a good compromise to help plan effective public health measures.

## Supplementary Material

Supplemental Material
