# Supplementary material for: A comparative analysis exposes an amplification delay distinctive to SARS-CoV-2 Omicron variants of clinical and public health relevance
Source: Emerg Microbes Infect. 2022 Dec 24;12(1):2154617. doi: 10.1080/22221751.2022.2154617 (PMC9793939; doi:10.1080/22221751.2022.2154617)
Supplement: Supplemental Material [file TEMI_A_2154617_SM8442.zip › Brown_etal_SM_11182022_EMI (1).docx]

**Additional Supplementary Information**

**A comparative analysis exposes an amplification delay distinctive to SARS-CoV-2 Omicron variants of clinical and public health relevance**

Brown K.L.^a,h^, Ceci A.^b^, Roby C.^b^, Briggs R.^b^, Ziolo D.^f^, Korba R.^g^, Mejia R.^g^, Kelly S.T.^g^, Toney D.^g^, Friedlander M.J.^d^, Finkielstein C.V. ^b,c,e,h,#^

^a^Virginia Tech Carilion School of Medicine, Virginia Tech, Roanoke, VA, USA

^b^Molecular Diagnostics Laboratory, Fralin Biomedical Research Institute at VTC, Virginia Tech, Roanoke, VA, USA

^c^Integrated Cellular Responses Laboratory, Fralin Biomedical Research Institute at VTC, Virginia Tech, Roanoke, VA, USA

^d^Fralin Biomedical Research Institute at VTC, Virginia Tech, Roanoke, VA, USA

^e^Department of Biological Sciences, Virginia Tech, Blacksburg, VA, USA

^f^ZC Lab Services, Greenacreas, FL, USA

^g^Molecular Detection and Characterization, Department of General Services, Division of Consolidated Laboratory Services, Richmond, VA, USA

^h^Center for Zoonotic and Arthropod-borne Pathogens, Virginia Tech, Blacksburg, VA, USA

**Description of Additional Supplementary Files**

**Supplementary Data 1.** Computer codes for the analysis of SARS-CoV-2 sequences.

**Description of Additional Supplementary Material**

**Supplementary Table 1.** Sequence of primers used for RT-qPCR amplification.

**Supplementary Table 2.** Oligonucleotides used for rapid mutation sequence analysis.

**Supplementary Figure 1.** Amplification of SARS-CoV-2 templates using N and S assay primers.

**Supplementary Figure 2.** Amplification comparisons for SARS-CoV-2 templates using different sets of E primers.

**Supplementary Figure 3.** Distribution of Cq values for N and S genes across variants over time.

**Supplementary Material Legends**

**Supplementary Table 1.** *Sequence of primers used for RT-qPCR amplification.* Primers [denoted as forward (f) and reverse (r) in the table] were designed using information deposited in the NCBI site (<https://www.ncbi.nlm.nih.gov/>). Specifically, GenBank accession number: MT039890.1 (for SARS-CoV-2 complete genome, Feb. 11^th^, 2020), *S* gene region: 21563..25384, *E* gene region: 26245..26472, *N* gene region: 28274..29533. Control primers for the housekeeping gene were retrieved from the Department of Health and Human Services, Centers for Disease Control and Prevention (CDC) bulletin, Atlanta, GA, entitled “2019-Novel Coronavirus (2019-nCoV) Real-time rRT-PCR Panel Primers and Probes” document from the Division of Viral Diseases (<https://www.cdc.gov/coronavirus/2019-ncov/lab/rt-pcr-panel-primer-probes.html>) on January 24, 2020.

**Supplementary Table 2.** *Oligonucleotides used for amplification and rapid mutation sequence analysis.* Primer sequences were derived from ARTIC V3 and V4.1 nCov-2019 primers. Customized oligonucleotides were designed using the PrimerQuest^TM^ tool (Integrated DNA Technologies) to generate amplicons of ~350nt. (*) Position of primers within the SARS-CoV-2 genome was based on the wild type isolate from Wuhan (Accession: MN908947.3)

**Supplementary Figure 1.** *Amplification of SARS-CoV-2 templates using N and S assay primers.* **A**, Schematic representation of a standard RT-qPCR amplification curve obtained by using a unique set of primers and either a matched template (red) or mismatched (black). Dashed lines indicate the slope of each curve from which overall efficiency is calculated. The area under the threshold shows the impact of the annealing mismatch in delaying early amplification. Amplification curves (fluorescence *vs.* cycle number) were obtained using the *Nf/Nr* or *Sf*/*Sr* set of primers and serial dilutions of the synthetic Delta (**B** and **D**, respectively) or Omicron (**C** and **E**, respectively) templates (200,000, 40,000, 8,000, 1,600, 320, or 64 copies/μl). All samples were run in triplicate and analysis was performed using the default settings in the CFX Maestro Software (BioRad).

**Supplementary Figure 2.** *Amplification comparisons for SARS-CoV-2 templates using different sets of E primers.* Background-adjusted fluorescence curves generated using the CFX Maestro Software (BioRad) obtained when using the *Ef*/*Er* set of primers (blue) and serial dilutions of Delta (**A**) and Omicron (**B**) synthetic templates (at 200,000, 40,000, 8,000, 1,600, 320, or 64 copies/μl). Amplification curves generated for Delta (**A**) and Omicron (**B**) using the following set of primers: *E-OM-1*/*Er*, *E-OM-2*/*Er*, *E-WuOM-1*/*Er*, *E-WuOM-2*/*Er* are depicted for each dilution in orange. All samples were run in triplicate and analysis was performed using the default settings in the CFX Maestro Software (BioRad).

**Supplementary Figure 3.** *Distribution of Cq values for N and S genes across variants over time.* 16,351 positive samples were reported between January 1,2021 and July 7, 2022. Distribution of Cq values for the *N* and *S* genes are shown for Delta (blue) and Omicron (red) over time (**A**). All other variants’ N and S Cq values are represented by a gray circle. A solid black line displays average Cq *N* and *S* values (**A** and **B**) and the shaded area shows 95% confidence intervals. **C**, Mean differences between *S* and *N* Cq values for positive samples tested within the same time frame. Error bars represent SD.
