# Supplementary material for: A comparative analysis exposes an amplification delay distinctive to SARS-CoV-2 Omicron variants of clinical and public health relevance
Source: Emerg Microbes Infect. 2022 Dec 24;12(1):2154617. doi: 10.1080/22221751.2022.2154617 (PMC9793939; doi:10.1080/22221751.2022.2154617)
Supplement: Supplemental Material [file TEMI_A_2154617_SM8442.zip › Sup_Table_1_11162022.docx]

**Supplementary Table 1**

| **Primer name** | **Sequence (5’ to 3’)** | **Target gene** | **SARS-CoV-2**  **Gene product** |
| --- | --- | --- | --- |
| N forward (*Nf*) | GCTGCAATCGTGCTACAACT | *N* gene | nucleocapsid protein |
| N reverse (*Nr*) | TGAACTGTTGCGACTACGTG |  |  |
|  |  |  |  |
| E forward (*Ef*) | TTCGGAAGAGACAGGTACGTT | *E* gene | Small envelope protein |
| E reverse (*Er*) | CACACAATCGATGCGCAGTA |  |  |
|  |  |  |  |
| S forward (*Sf*) | GCTGCAGCTTATTATGTGGGTTATC | *S* gene | Spike surface glycoprotein |
| S reverse (*Sr*) | GAGGGTCAAGTGCACAGTCTACAG |  |  |
|  |  |  |  |
| RPP30 forward (*RPP30f*) | AGATTTGGACCTGCGAGCG | *RPP30* gene | Human Ribonuclease P (RNase P) protein |
| RPP30 reverse (*RPP30r*) | GAGCGGCTGTCTCCACAAGT |  |  |
