# Supplementary material for: A comparative analysis exposes an amplification delay distinctive to SARS-CoV-2 Omicron variants of clinical and public health relevance
Source: Emerg Microbes Infect. 2022 Dec 24;12(1):2154617. doi: 10.1080/22221751.2022.2154617 (PMC9793939; doi:10.1080/22221751.2022.2154617)
Supplement: Supplemental Material [file TEMI_A_2154617_SM8442.zip › Sup_Table_2_11162022.docx]

**Supplementary Table 2**

| **Amplicon**  **name** | **Forw./**  **Rev.** | **Sequence (5’ to 3’)** | **Primer positions (*)** | **Amp. size** |
| --- | --- | --- | --- | --- |
| Nucleocapsid  537-909 | Forward | CAGTCAAGCCTCTTCTCGTTC | 28810-28830 | 373 |
|  | Reverse | TTGCGGCCAATGTTTGTAATC | 29182-29162 |  |
|  |  |  |  |  |
| Spike-6C | Forward | ATATTCTAAGCACACGCCTATT | 22171-22192 | 210 |
|  | Reverse | CTAGGTTGAAGATAACCCACATAA | 22380-22357 |  |
|  |  |  |  |  |
| Spike ARTIC76V4.1 | Forward | ATGTCTATGCAGATTCATTTGTAATTAGAGGT | 22743-22774 | 399 |
|  | Reverse | GTCCACAAACAGTTGCTGGTG | 23141-23121 |  |
|  |  |  |  |  |
| Spike art-75om-v3 | Forward | AGAGTCCAACCAACAGAATCTATTGT | 22517-22542 | 386 |
|  | Reverse | ACCACYAACCTTAGAATCAAGTTTGT | 22903-22878 |  |
|  |  |  |  |  |
| Spike ARTIC-71-V3 | Forward | ACAAATCCAATTCAGTTGTCTTCCTATTC | 21358-21386 | 385 |
|  | Reverse | TGGAAAAGAAAGGTAAGAACAAGTCCT | 21743-21717 |  |
|  |  |  |  |  |
| Orf1a/b ARTIC-9V3 | Forward | TTCCCACAGAAGTGTTAACAGAGG | 2505-2528 | 399 |
|  | Reverse | GAGAGCATCTGCCACAACACAG | 2902-2881 |  |
|  |  |  |  |  |
| Spike ARTIC-78-V3 | Forward | CAACTTACTCCTACTTGGCGTGT | 23444-23466 | 404 |
|  | Reverse | TGTGTACAAAAACTGCCATATTGCA | 23847-23823 |  |
|  |  |  |  |  |
| Envelope art-87-v3 | Forward | CGACTACTAGCGTGCCTTTGTA | 26198-26219 | 392 |
|  | Reverse | ACTAGGTTCCATTGTTCAAGGAGC | 26590-26567 |  |
