# Supplementary figures and images for: A comparative analysis exposes an amplification delay distinctive to SARS-CoV-2 Omicron variants of clinical and public health relevance
Source: Emerg Microbes Infect. 2022 Dec 24;12(1):2154617. doi: 10.1080/22221751.2022.2154617 (PMC9793939; doi:10.1080/22221751.2022.2154617)

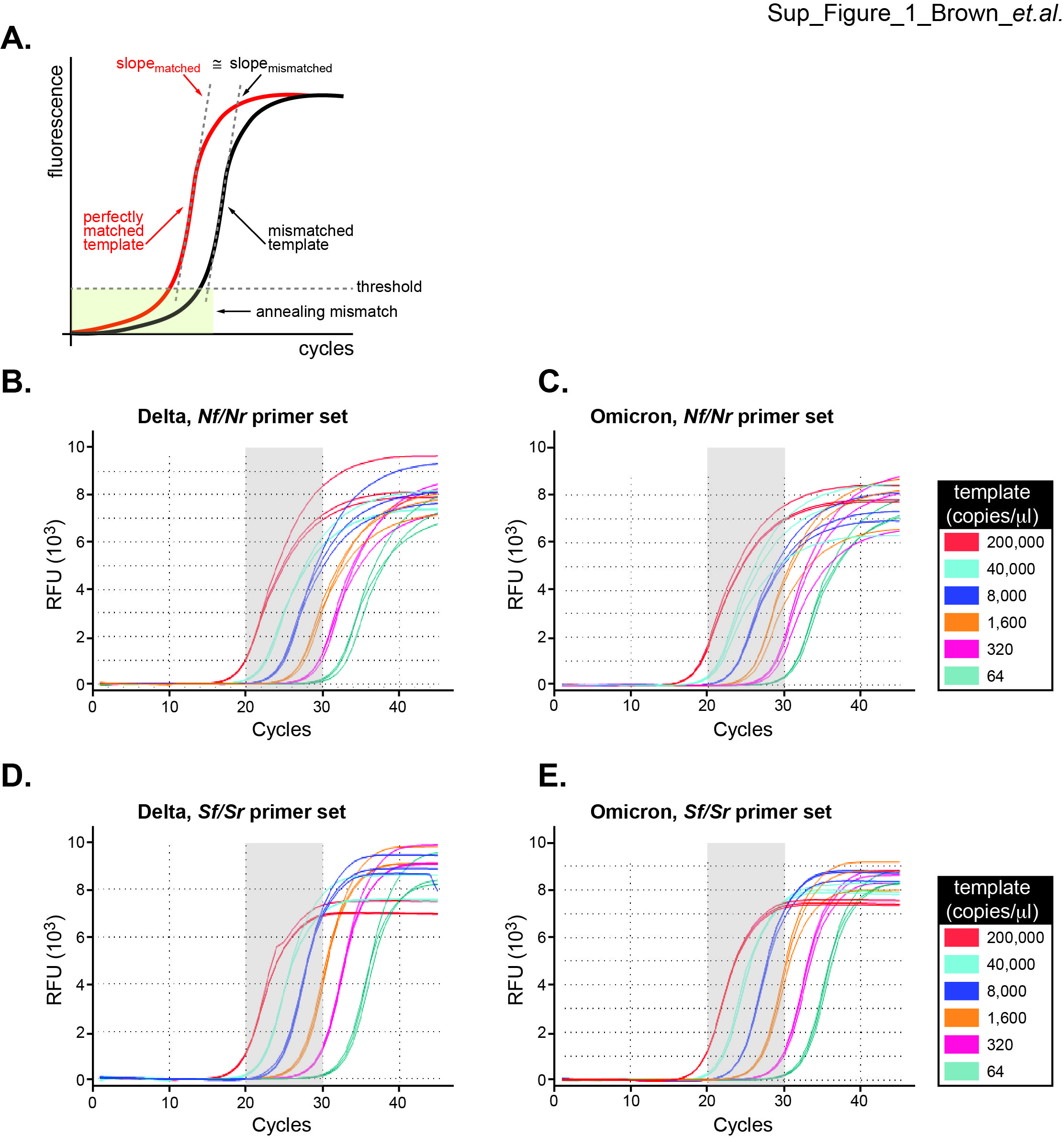

Supplement: Supplemental Material [file TEMI_A_2154617_SM8442.zip › Sup_Fig_1_11162022.jpg]

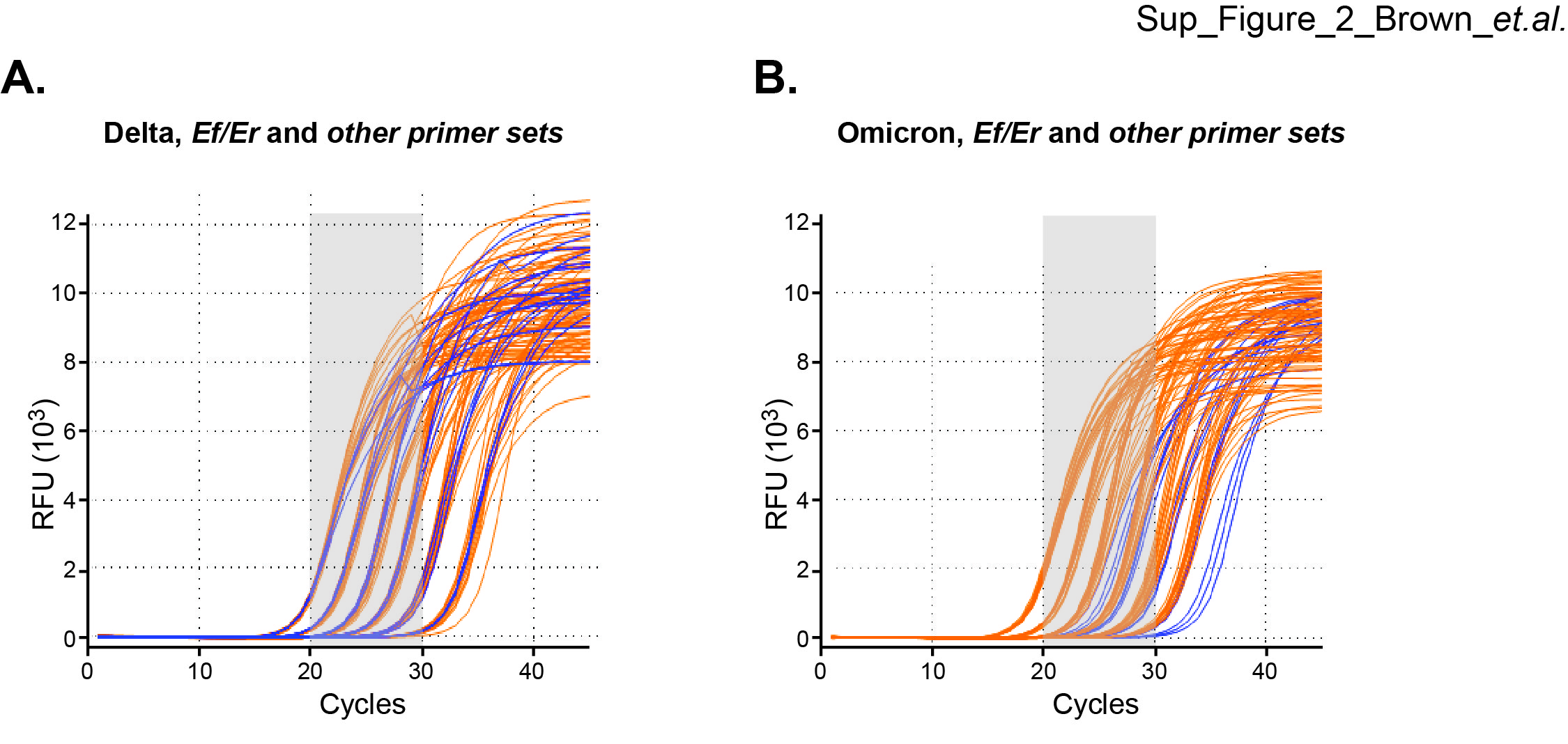

Supplement: Supplemental Material [file TEMI_A_2154617_SM8442.zip › Sup_Fig_2_11162022.jpg]

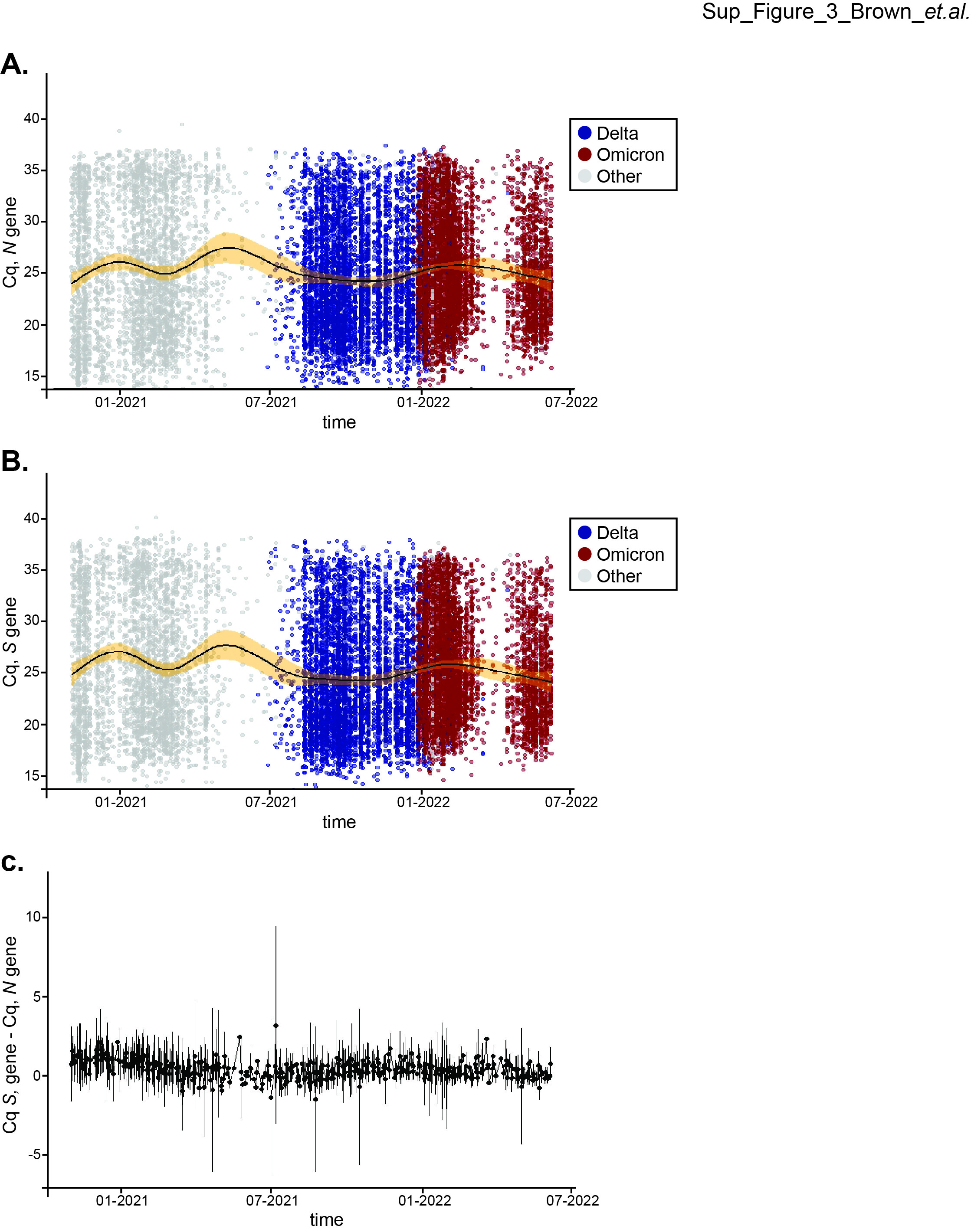

Supplement: Supplemental Material [file TEMI_A_2154617_SM8442.zip › Sup_Fig_3_11162022.jpg]
